# Social Modulation of Stress Reactivity and Learning in Young Worker Honey Bees

**DOI:** 10.1371/journal.pone.0113630

**Published:** 2014-12-03

**Authors:** Elodie Urlacher, Ingrid S. Tarr, Alison R. Mercer

**Affiliations:** Department of Zoology, 340 Great King Street, PO Box 56, Dunedin 9054, New Zealand; The University of Queensland, Australia

## Abstract

Alarm pheromone and its major component isopentylacetate induce stress-like responses in forager honey bees, impairing their ability to associate odors with a food reward. We investigated whether isopentylacetate exposure decreases appetitive learning also in young worker bees. While isopentylacetate-induced learning deficits were observed in guards and foragers collected from a queen-right colony, learning impairments resulting from exposure to this pheromone could not be detected in bees cleaning cells. As cell cleaners are generally among the youngest workers in the colony, effects of isopentylacetate on learning behavior were examined further using bees of known age. Adult workers were maintained under laboratory conditions from the time of adult emergence. Fifty percent of the bees were exposed to queen mandibular pheromone during this period, whereas control bees were not exposed to this pheromone. Isopentylacetate-induced learning impairments were apparent in young (less than one week old) controls, but not in bees of the same age exposed to queen mandibular pheromone. This study reveals young worker bees can exhibit a stress-like response to alarm pheromone, but isopentylacetate-induced learning impairments in young bees are suppressed by queen mandibular pheromone. While isopentylacetate exposure reduced responses during associative learning (acquisition), it did not affect one-hour memory retrieval.

## Introduction

Honey bees are active defenders of their colony. Their stinging and biting behavior is very effective at repelling intruders, even intruders as large as bears [1]. Guard bees initiate colony-level responses by identifying threats and recruiting nest mates for collective defense [2]. Recruitment is achieved through chemical communication. Volatile chemicals (pheromones) released by guards ‘sound the alarm’ and attract recruits to the entrance of the colony [3]–[5]. Interestingly, young bees normally stay within the hive and do not participate in colony defense [1]–[2]. However, the bees that are successfully recruited by alarm pheromone display changes in behavior and physiology that resemble stress responses. These changes include increased agitation, a rise in respiratory rates [2], and enhanced aggressiveness [5]–[7]. Responses occur at the molecular level also with the induction of immediate early genes [8]. Recently, Urlacher and colleagues have shown that stress induced by exposing foragers to alarm pheromone, or to its main component isopentyl acetate (IPA), decreases appetitive learning in these bees [9]. We were interested to determine whether this IPA-induced stress response, like the recruitment responses to alarm pheromone, is age dependent.

The probability that a honey bee will respond to a stimulus, or perform a specific behavior is strongly influenced by age. Behavioral maturation is prominent in bees, and division of labor within honey bee colonies is based on this temporal polyethism [10]. When worker bees first emerge as adults, they perform tasks such as cell cleaning and capping. After a few days they shift to other in-hive tasks, such as feeding developing larvae (nursing) and tending the queen. As they age further, workers move to the periphery of the colony where they build comb, guard the hive entrance and handle incoming nectar and pollen collected by the oldest workers in the colony, the foragers. As young bees are reported to show little response to alarm pheromone [11] and do not participate in colony defense, we hypothesize that appetitive learning in young bees would not be affected by IPA.

Under normal colony conditions the behavior, particularly of young workers, is strongly influenced by pheromones released by the queen bee. Queen mandibular pheromone (QMP), for example, which helps attract young attendants to the queen and inhibits ovary development in workers, also slows the behavioral maturation of worker bees [12]–[14]. QMP has also been found to suppress aversive learning in young bees and specifically, their ability to associate odors with electric shock [15]. This is of interest in this context because electric shock, like alarm pheromone, is a stimulus that induces stress-like responses in bees [16]. QMP's effects on aversive learning could potentially be explained in part by a reduction in stress reactivity in young workers. If IPA is found to reduce appetitive learning in young bees, the possibility that QMP reduces stress reactivity will be explored by determining whether exposure to QMP influences stress related responses induced by IPA.

The effect of QMP on aversive learning is age-dependent, in 15-day old bees aversive learning appeared to be unaffected by QMP exposure, whereas in bees around 6 days of age aversive learning can be completely blocked by this pheromone [15]. For this reason, effects of age will be examined also in this study.

The goals of the current study are twofold; to determine whether IPA induces learning deficits in young worker bees and if so, to investigate whether this stress-related response is modulated by QMP.

## Materials and Methods

### Animals

All bees were collected from hives housed at the University of Otago, New Zealand, from colonies typically containing several tens of thousands of worker bees, brood and a naturally-mated queen. All experiments were performed on *Varroa*-free bees, as the parasite had not yet infested this region at the time of the experiments. Bees performing tasks characteristic of different age groups (cell cleaning, guarding or returning from foraging) were collected directly from hives. Cell cleaning and guarding are typically performed by young and middle-aged bees respectively, whereas foraging is normally performed by the oldest bees in the colony [10], [17]–[19]. Very young bees can be identified by their lighter color, their relatively slow movements and their poorly-developed flying skills. Bees showing these characteristics that had their heads in empty cells were classified as cell cleaners. Bees that were present at the hive entrance prior to any disturbance and reacted to forceps with biting, raised forelegs and alarm pheromone emission were classified as guards. Bees returning to the hive with full pollen baskets were identified as pollen foragers.

To collect bees of known age, brood frames from the hives were placed under constant darkness in a humidified incubator at 33°C. Each sampling day, during a fixed 4-hour time window (12:00 h–16:00 h) adult bees were collected as they emerged from brood cells. Newly-emerged bees were marked with paint on the thorax so that their age could be determined at a later date, and placed in cages (11×9.5×7.5 cm) containing approximately 50 bees of mixed age. To avoid bias due to seasonal variation, bees were raised to different ages in parallel rather than sequentially. Half of the cages contained a strip impregnated with QMP (BeeBoost, PheroTech 2.5 queen equivalent per cage, changed weekly [20]). Cages with and without QMP strips were maintained in separate incubators (31°C, 70% humidity). Each cage contained a sheet of wax and bees were provided also with food (pollen, honey and sucrose mixture) and water *ad libitum.*

### Stress protocol

Bees were prepared for behavioral experiments immediately after being collected either from the hive (cell cleaners, guards and foragers), or from cages when they were 2-, 4-, 6-, 8- or 16-days old. Bees were handled as described in [9]. Briefly after being cooled down on ice for a few minutes, they were individually harnessed in holders that allowed free movements of the mouthparts and antennae. The bees were starved for 4 to 5 hours to enhance motivation, then exposed for 30 minutes either to IPA or vehicle alone (oil) before undergoing appetitive olfactory conditioning. For IPA exposure, the same protocol was used as described elsewhere [9]; harnessed bees were placed individually in a 35 ml glass vial containing a small piece of filter paper soaked with 25 µl of IPA (24% in paraffin oil, Sigma-Aldrich). Controls were exposed in the same way to vehicle alone (paraffin oil). The closed vials containing the bees being treated with IPA were placed in an air exhaust system throughout the 30 min of exposure period to avoid contamination between groups. Following IPA (or oil) exposure, bees were allowed to recover for 30 min before conditioning.

### Sucrose responsiveness

Age, QMP exposure and IPA can all affect sucrose responsiveness [7], [9], [28]–[30], [32]. For this reason, only bees that responded to sucrose stimulation consistently throughout each experiment were included in the analysis. While sucrose response thresholds were not measured directly in our experiments, the percentage of bees in each group that failed to respond consistently to 50% sucrose during conditioning, and/or in the memory tests, was recorded. In the group exposed to neither pheromone, 11% of the bees were discarded. In bees exposed to IPA, 21% of the bees were discarded. In the group reared with QMP, 18% failed to respond, whereas 22% of bees exposed to both pheromones were discarded.

### Appetitive olfactory conditioning

The proboscis conditioning paradigm developed by Bitterman and colleagues [21] was used in this study. Each bee received 3 paired presentations of an odor (1-nonanol, 5 µl, pure, Sigma-Aldrich) and sucrose (50%, w/w in water) with a 10 minute interval between each conditioning trial. Prior to conditioning, the conditioned stimulus (CS, 1-nonanol), elicited no response, whereas 50% sucrose (the unconditional stimulus US), applied to the antennae elicited a reflexive proboscis extension response. Any bee that failed to respond reflexively to the US was discarded. Odors were delivered by means of a 20 ml syringe containing a piece of filter paper soaked with 5 µl of pure odorant. To avoid odor contamination, conditioning trials were performed in front of an air exhaust system. Bees were placed in the setup 15 seconds before the CS was presented, for 4 seconds. Sucrose was presented 3 seconds after odor onset and was delivered first to the antennae to elicit proboscis extension and then to the proboscis. Bees were allowed to lick the sucrose solution for 3 seconds. After conditioning, bees that show learning extend their proboscis in response to the odor before the reward is delivered (conditioned response CR). The percentage of bees showing the conditioned response is used as a measure of learning. This assay was used to measure the effect of IPA-induced stress on appetitive learning performances.

### Memory test

One hour after the end of the last conditioning trial, bees were placed again in the learning area and presented with the CS alone, without reinforcement. The percentage of bees displaying a conditioned response (CR) was recorded. At the conclusion of each experiment, the proboscis extension reflex was tested again. Bees that failed to respond to sucrose stimulation of the antennae were discarded from the experiment.

### Statistics

The data were analysed with generalised linear mixed effects modelling (GLMM) using the R package lme4 [22]. To improve the convergence of the acquisition model, only the second and third trials were analysed (as all groups showed the CR 0% of the time on the first trial). The predictor “Trial” was rearranged such that the third trial was set to zero (as Trial 3), thus becoming the intercept of the model. In this way, model predictors provided information about the probability of responses on the last trial. Slopes for the response of each individual bee (Trial|Subject) across the two trials were included as random effects in this GLMM.

In the first experiment examining learning performance of bees belonging to different behavioral castes, GLMM was used to investigate the fixed effects of conditioning trial (Trial), IPA exposure and Caste, as well as interaction terms for pheromone treatment and Caste. The interaction term was included, as it markedly improved the convergence of the model.

GLMM was also used to investigate the fixed effects of Trial, Age, IPA exposure and QMP-rearing in laboratory-raised bees of different age, as well as all interaction terms for pheromone treatment and age (IPA exposure × QMP-rearing × Age). The interaction term was included, as it markedly improved the convergence of models. Thus, the effects of these variables over the third trial of the conditioning period could be assessed. With these estimates of the final effects of these variables, comparisons could be made both within and between treatment groups.

The probability of bees displaying a conditioned response one hour after the conclusion of conditioning, during the retention test, was examined for both experiments also using a GLM in which all possible terms for pheromone exposure and age (or caste) were included, as in the GLMM.

We also used GLM to analyse the effect of pheromone treatments on two broad age groups; young bees (≤6 d) and bees 8-days and older. The third conditioning trial only was examined for this purpose. Data obtained from bees 6 days of age or less were pooled and compared to pooled data from bees 8 days of age or more. The interaction of three factors (IPA exposure × QMP-rearing × Age) was included.

All statistics were generated in R [23], and results are presented as coefficients ± standard error.

## Results

To begin, we tested the learning performance of bees collected from a queen-right colony. Bees were grouped according to the tasks they were performing at the time of capture. Three groups were examined; cell cleaners, guards and foragers. Cell cleaners are generally 1–5 days old [10], [17]–[19], and were assumed to be the youngest bees in this experiment. Guards are typically middle-age bees (around 15-day olds of age) whereas foragers are generally more than 3 weeks old and the oldest workers in a colony [10], [17]–[19]. We looked for IPA-induced learning deficits in these 3 behavioral castes.

**Figure 1:**
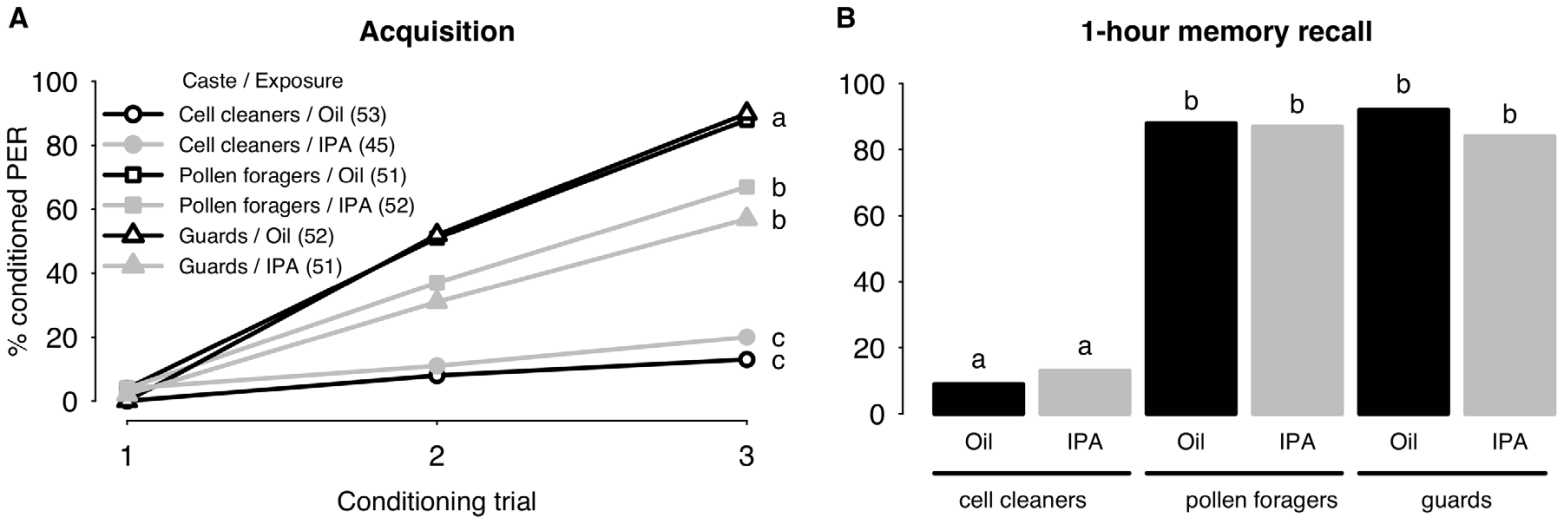
Learning and 1-h memory recall in different behavioral castes exposed to IPA or to Oil (control). 1A. Acquisition curves show changes in the percentages of bees displaying the conditioned proboscis extension response (PER) over three successive conditioning trials. Letters (a, b, c) indicate significant differences between groups in the final conditioning trial (refer to text for p values). Groups that share a letter are not significantly different. The number of bees in each group is indicated in brackets. 1B. 1-hour memory recall. Response levels differ significantly in groups with a different letter (refer to text for p values).

As can be seen in Figure 1A, differences in learning performances were detected between bees exposed to oil and IPA (black and grey lines respectively in Figure 1A). However, while pollen foragers and guards had reduced CR probabilities when exposed to the alarm pheromone (pollen foragers −0.93±0.31, p<0.01; guards −1.31±0.31, p<0.001), IPA had no effect on the probability of responses in cell cleaners (0.46±0.44, p = 0.29). Accordingly there was a significant interaction between the factors “Behavioral caste” and “IPA exposure" when comparing nurses to guards (−1.77±0.54, p<0.01) and pollen foragers (−1.39±0.54, p<0.01). Response probabilities were affected by behavioral caste. The probability of guards and pollen foragers displaying a conditioned response was similar (0.03±0.32, p = 0.92), but both of these castes showed stronger learning performances than cell cleaners (guards vs. cleaners 3.31±0.40, p<0.01; foragers vs. cleaners 3.29±0.40, p<0.001). In all three behavioral groups, memory retrieval 1-h after the last conditioning trial was unaffected by IPA (Figure 1B); the probability of IPA-treated bees displaying a CR in the memory retention test was similar to that of bees treated with oil (cell cleaners 0.39±0.64, p = 0.54; pollen foragers −0.15±0.59, p = 0.80; guards −0.80±0.65, p = 0.22). However, differences between cell cleaners, guards and pollen foragers persisted (cleaners vs. guards 4.75±0.70, p<0.001; cleaners vs. pollen foragers 3.88±0.59, p<0.001). This experiment revealed differences in learning performance between behavioral castes and differences also in their susceptibility to IPA-induced learning impairments. Next we examined whether these effects were age dependent, and whether IPA-induced stress responses might be modulated by QMP.

**Figure 2:**
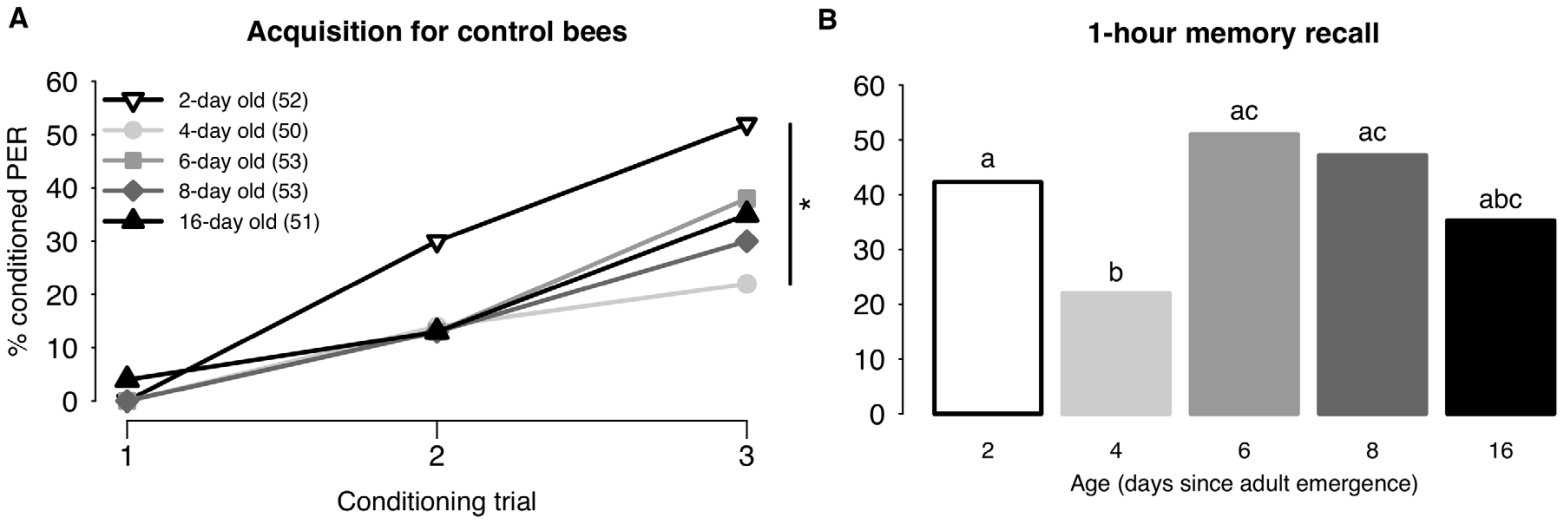
Learning and 1-h memory recall of 2- to 16-day old laboratory-raised bees. Bees presented here were exposed to neither IPA nor QMP. 2A. Acquisition curves for 2-, 4-, 6-, 8- and 16-day old bees. Acquisition curves show changes in the percentages of bees responding to the conditioned stimulus over 3 successive conditioning trials. Asterisk indicates a significant difference between groups in the final conditioning trial (refer to text for p values). The number of bees in each group is indicated in brackets. 2B. 1-hour memory recall. Response levels differ significantly in groups with a different letter (refer to text for p values).

Learning behavior and responsiveness to IPA was examined in 2-, 4-, 6-, 8- and 16-day old bees maintained under laboratory conditions. The learning performances of bees of different ages exposed to oil alone (controls) are shown in Figure 2A. Overall, the probability of control bees responding to the conditioned stimulus increased significantly between the second and third conditioning trials (7.42±0.42, p<0.001; Figure 2A). However, not all age groups performed equally well; CR probability was affected by age, with 2-day olds performing better than 4-day olds (−0.97±0.42, p<0.05), but not differently from other ages (6 d −0.41±0.40, p = 0.30; 8 d −0.68±0.41, p = 0.09; 16 d −0.66±0.41, p = 0.11). Compared to guards and foragers, memory retrieval in cage-reared bees was relatively poor, particularly in 4-day old bees (Figure 2B), which had lower response probabilities than 2-, 6- and 8- day olds (2 d 0.89±0.44, p<0.05; 6 d 1.30±0.44, p<0.01; 8 d 1.15±0.44, p<0.01), but not lower than 16-day olds (0.63±0.45, p = 0.16).

The ability of IPA to induce a stress response (learning impairment) was examined in these 5 age groups. For each rearing condition (with or without QMP), learning was compared between oil-treated and IPA-exposed bees. For purposes of clarity, learning performances of bees of different ages are presented separately.

**Figure 3:**
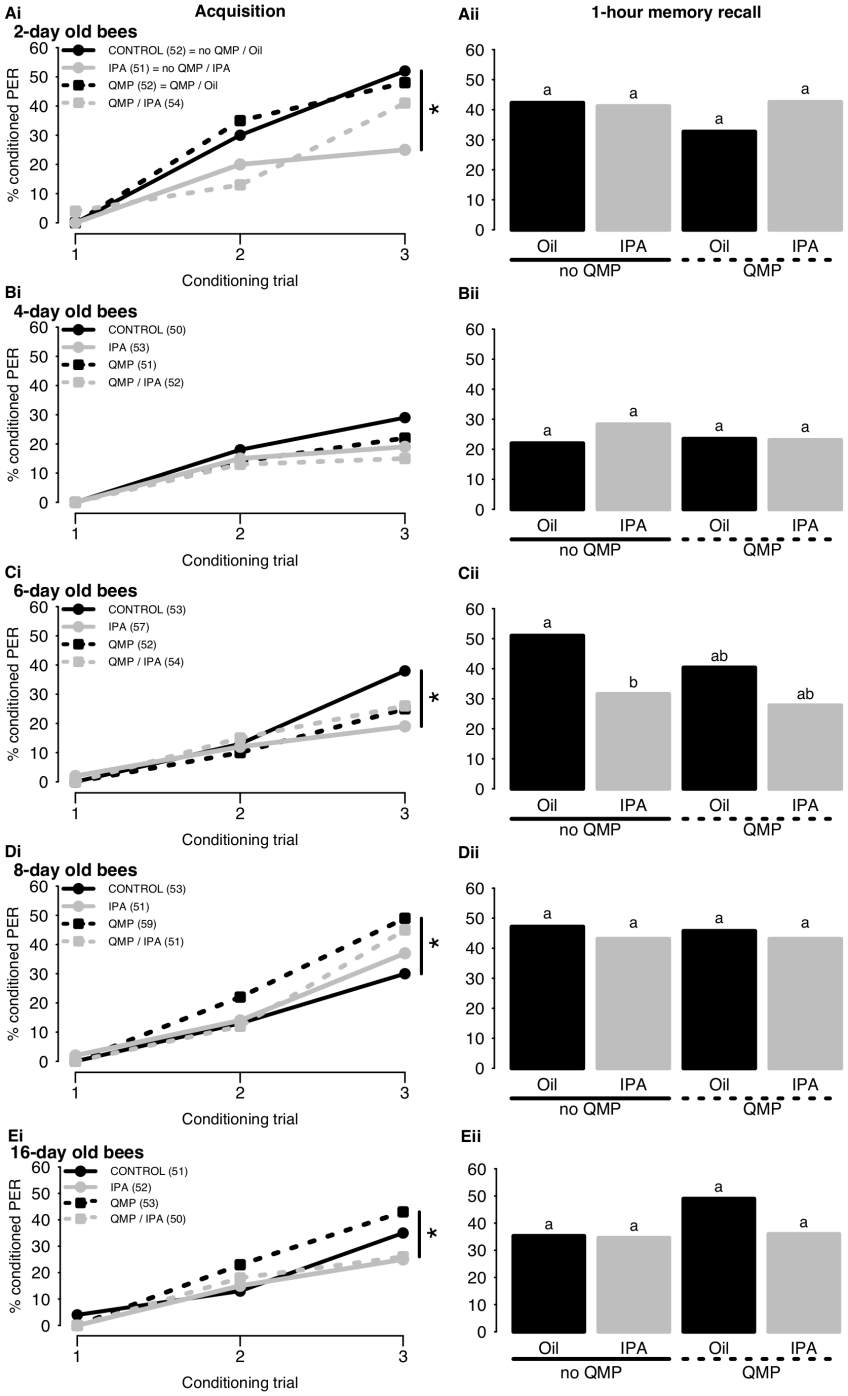
Effects of IPA and QMP on learning and 1-h memory recall in 2 to 16-day old bees (A: 2-day old, B: 4-day old, C: 6-day old, D: 8-day old, E: 16-day old). 3i. Acquisition curves show changes in the percentages of bees responding to the conditioned stimulus over 3 successive conditioning trials. Asterisk indicates a significant difference in the third conditioning trial (refer to text for p values). The number of bees in each group is indicated in brackets. 3ii. 1-hour memory recall. Response levels differ significantly in groups with a different letter. For purposes of clarity, comparisons between treatments are made only within, not between age groups (refer to text for p values).

Response probability was significantly higher in 2-day old control bees exposed to oil alone (solid black line in Figure 3Ai) than in bees of the same age treated with IPA (−1.01±0.43, p<0.05, solid grey line in Figure 3Ai). However, IPA's effects were blocked in 2-day old bees that had been raised from the time of adult emergence with queen pheromone. IPA had no effect on the probability of responses in bees reared with QMP (−0.18±0.40, p = 0.64, dashed lines). Unlike IPA, queen pheromone alone did not alter the CR probability of oil-exposed bees (Control vs. QMP 0.02±0.40, p = 0.96) but it did tend to improve it in 2-day old bees exposed to IPA (IPA vs. QMP/IPA 0.80±0.43, p = 0.06). In the memory retention tests (Figure 3Aii), response probabilities of 2-day old bees were similar across all four groups, irrespective of whether bees had been treated with IPA and/or QMP (Control vs. IPA 0.02±0.40, p = 0.96; QMP vs. QMP/IPA 0.42±0.40, p = 0.29; Control vs. QMP −0.35±0.41, p = 0.39; IPA vs. QMP/IPA 0.06±0.40, p = 0.88).

Learning overall was poor in 4-day old bees and was not affected by IPA (Figure 3Bi). IPA exposure had effect on neither controls (Control vs. IPA −0.20±0.47, p = 0.68 solid lines) nor QMP-reared bees (QMP vs. QMP/IPA −0.52±0.54, p = 0.35, dashed lines). Maintaining bees from the time of adult emergence with QMP also had no effect on response probabilities in this age group (Control vs. QMP −0.28±0.49, p = 0.57; IPA vs. QMP/IPA −0.86±0.56, p = 0.12). All four groups of 4-day old bees also showed very similar percentages of CR in the memory retention tests (Figure 3Bii, Control vs. IPA 0.34±0.46, p = 0.46; QMP vs. QMP/IPA −0.02±0.47, p = 0.96; Control vs. QMP 0.09±0.48, p = 0.85, IPA vs. QMP/IPA −0.27±0.45, p = 0.54).

In 6-day old bees, IPA treatment altered learning and QMP affected this modulation (Figure 3Ci). Alarm pheromone exposure decreased CR probability in bees raised without QMP (−1.13±0.46, p<0.05), but interestingly, no effect of IPA was evident in bees reared with QMP, these bees had very similar responses probabilities on the final trial (−0.10±0.47, p = 0.85). Therefore, QMP rescued 6-day old bees from the inhibitory effects of IPA, as could be observed in 2-day olds. In this age group, QMP treatment seemed to decrease CR probability, but this result did not reach significance (Control vs. QMP −0.73±0.44, p = 0.09). In the memory test, the difference in CR between bees exposed to neither pheromone and bees exposed to IPA faded slightly, but still reaching significance (−0.81±0.40, p<0.05, Figure 3Cii). QMP-reared groups showed intermediate CR on the recall test (Control vs. QMP −0.43±0.39, p = 0.28), and were not affected by IPA exposure (−0.30±0.40, p = 0.45).

Responses to IPA and QMP in 8- and 16-day old bees were markedly different from those observed in the younger age groups tested (Figures 3D and 3E). In 8-day olds, exposure to IPA had no significant effect on learning, neither in controls (0.25±0.43, p = 0.56), nor in bees exposed to the queen pheromone (−0.05±0.39, p = 0.91, Figure 3Di). Moreover, in contrast to the younger age groups, response probabilities were enhanced by QMP in 8-day olds (black lines in Figure 3Di; 0.99±0.40, p<0.05), although not if bees were exposed to IPA (0.35±0.41, p = 0.39, grey lines). In the memory retention test (Figure 3Dii), no differences between the groups could be detected (Control vs. IPA −0.16±0.39, p = 0.81; QMP vs. QMP/IPA −0.38±0.38, p = 0.32; Control vs. QMP 0.21±0.38, p = 0.57, IPA vs. QMP/IPA 0.00±0.40, p = 1).

In 16-day old bees, in contrast to all other age groups, inhibitory effects of IPA were stronger in bees exposed to QMP (Figure 3Ei). While IPA exposure appeared to have a slight detrimental effect in control bees reared without queen pheromone, the effect was not significant (−0.50±0.45, p = 0.26). However, in 16-day old bees exposed to QMP from the time of adult emergence, IPA reduced appetitive learning significantly (−0.90±0.45, p<0.05), consistent with effects of IPA on guards and foragers collected from a queen-right colony (Figure 1A). In 16-day olds, as in 8-day olds, CR probabilities appeared to be enhanced by QMP. However, in this age group, QMP's effects were not statistically significant (Control vs. QMP 0.34±0.42, p = 0.42; IPA vs. QMP/IPA 0.05±0.46, p = 0.92). Differences observed during conditioning disappeared when the 1-hour memory of 16-day olds was tested (Figure 3Eii). Neither IPA (Control vs. IPA 0.00±0.41, p = 1; QMP vs. QMP/IPA −0.54±0.40, p = 0.18), nor QMP (Control vs. QMP 0.60±0.40, p = 0.13; IPA vs. QMP/IPA 0.06±0.41, p = 0.88) had a significant effect on the 1-hour memory recall in this age group.

**Figure 4:**
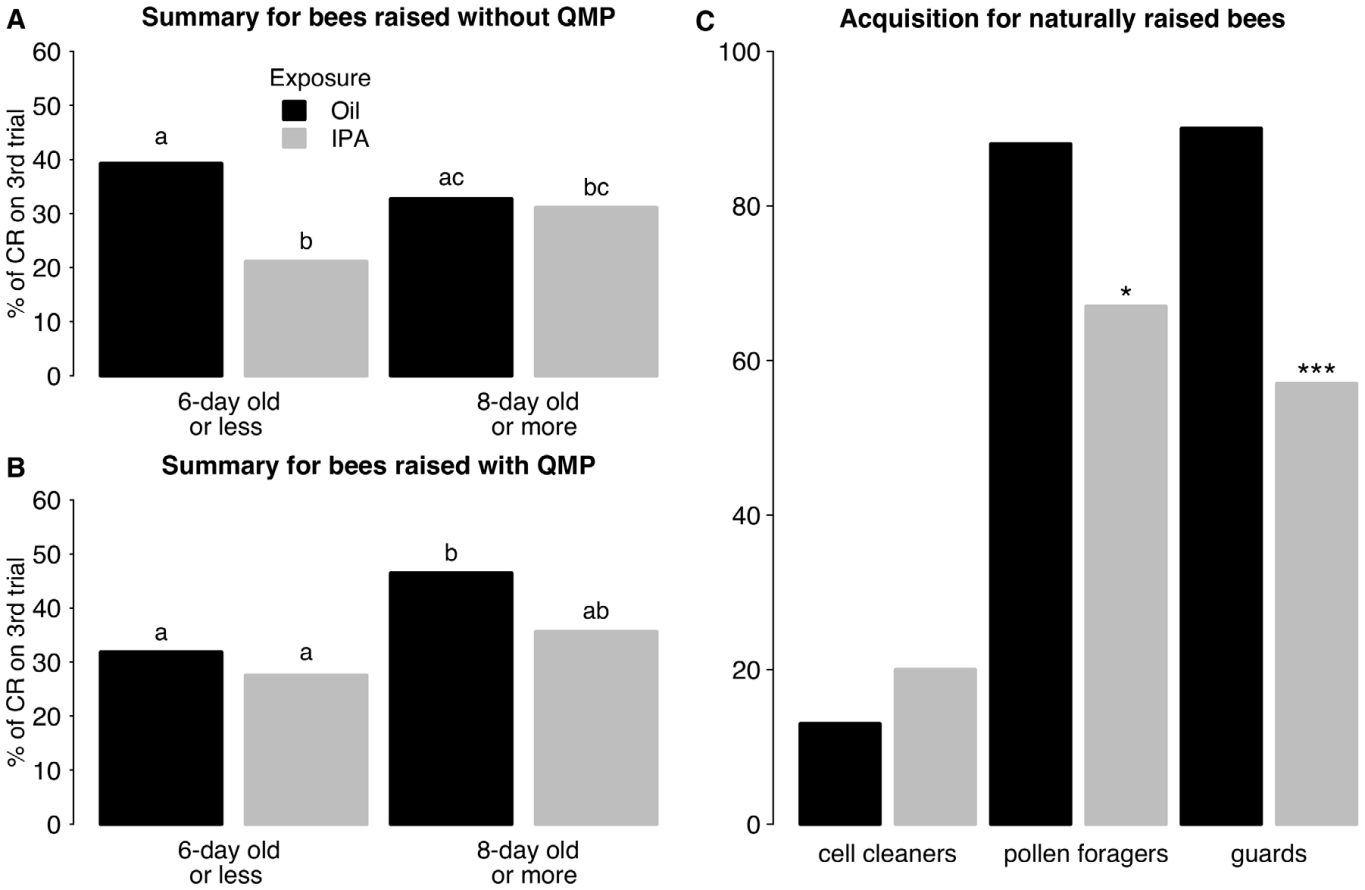
Comparison of laboratory- and naturally-raised bees. Learning performance in the third conditioning trial. Levels of conditioned responses in control bees (black bars) are compared with those of bees treated with IPA (grey bars). A, B. Bees raised under laboratory conditions. Data were pooled to form two age groups: bees less than 1 week old (2-, 4- and 6-day old bees) and bees 8- or 16-days old. Responses of bees raised without QMP (A) can be compared to those of bees raised with QMP (B). C. Responses of bees from a queen-right colony. A, B. Response levels differ significantly in groups with a different letter, comparisons are only made within each figure (refer to text for p values). C. Asterisks indicate significant differences between control and IPA-treated groups (* p<0.05, *** p<0.001).

Our results suggest that interactions between QMP and IPA are age-dependent. To examine the interactions more closely, we pooled the data obtained for 2-, 4- and 6- day old bees, and compared it to pooled data for 8- and 16-day olds. For simplicity, we chose to focus on responses in the last conditioning trial only in order to clarify the effect of QMP on IPA-induced learning deficits. In bees less than 1 week of age (2-, 4- and 6-day old bees) reared without QMP, IPA reduced significantly the level of conditioned responses observed in the third and final conditioning trial (Figure 4A, first two bars, −0.88±0.25, p<0.001). In marked contrast to this, IPA had no effect on learning performance in young bees that had been exposed to QMP (Figure 4B, first two bars, −0.21±0.25, p = 0.40). Moreover, in this age category, QMP did not affect learning (bees exposed to oil −0.32±0.24; to IPA 0.35±0.26, p = 0.18).

The pooled data show QMP's enhancing effect on the level of conditioned responses in older bees (compare Figures 4A and 4B, third bar, 0.58±0.28, p<0.05), an enhancement that is suppressed slightly in IPA-exposed bees (0.21±0.30, p = 0.48, Figure 3B). They also shows that for the older age groups examined, IPA failed to have any detrimental effect in bees reared without QMP (−0.07±0.30, p = 0.80, Figure 4A), whereas it had some tendency to do so in QMP-reared bees (−0.45±0.28, p = 0.11, Figure 4B). Responses of bees from a queen-right colony are included here for comparison (Figure 4C). The response probabilities of laboratory-raised bees are higher than those of cell cleaners but lower than those of pollen foragers and guards. Response probabilities in cell cleaners are similar to those observed in 4- and 6-day old bees, but lower than that observed in 2-day olds. IPA did not inhibit learning in cell cleaners (0.47±0.55, p = 0.39), nor in laboratory-raised young bees exposed from the time of adult emergence to QMP. Pollen foragers and guards showed the strongest learning performance of all the bees tested in this study. Interestingly, even when the last conditioning trial alone was analysed, IPA could be seen to reduce significantly the probability of responses in these two behavioral castes (pollen foragers −1.29±0.52, p<0.05; guards −2.04±0.55, p<0.001). A similar trend is apparent in the older age groups that were reared in the laboratory and exposed to QMP (8- and 16-day old bees pooled, Figure 4B), a trend that was significant when 16-day olds were analysed on their own (Figure 3Ei).

## Discussion

This study reveals three important findings. We show that bees as young as 2-days of age can display a stress response to IPA (impaired reward learning) similar in magnitude to responses displayed by guards and foragers. This is of interest because young bees are generally reported to respond less to alarm pheromone than older bees [11], [24]. Importantly in this context, we show that in young bees, effects of IPA on appetitive learning can be suppressed by QMP. This lends strong support to the hypothesis that QMP contributes to age-related shifts in worker bee reactivity to alarm pheromones described in earlier reports. Finally, our study reveals that while IPA is able to suppress responses to conditioned olfactory stimuli during memory acquisition, 1-hour memory remains intact.

### Multiple factors affect learning performance

Consistent with age-related changes in learning behavior described in earlier reports [25]–[29], learning performances were found to be stronger overall in guards and foragers than in the young bees tested in this study, including cell cleaners collected from a queen-right colony. Nonetheless, differences in the learning performances of 16-day old bees and guards were unexpected because these bees were assumed to be similar in age [10], [17]–[19]. However, learning performances can be influenced also by factors such as sucrose responsiveness [30], hormone titers [31] and motivation [32], and it is possible that the learning levels observed in this study were influenced by factors such as these. For instance hormone titers vary greatly during the first days after adult emergence [17], [33]–[34] and may account for the differences in learning performances between bees of different ages observed in the present study. Artificial rearing might also influence learning levels. For example, cage-reared bees were fed *ad libitum* which may have reduced their motivation to learn [35]. The absence of brood pheromone in the cages might also modify hormone titers in these artificially raised bees [36] and indirectly also, their learning behavior. Nonetheless, despite the artificial environment experienced by laboratory-reared bees, all of the age- and behavioral groups examined in this study showed significant learning and within each group, performances in the 1-hour memory test were highly consistent. Effects of IPA on learning behavior, described previously in foragers [9], were found to be remarkably robust also in this study.

### Young bees raised without QMP show strong reactivity to IPA

As young bees performing tasks within the colony generally ignore the “call to arms” signaled by the release of alarm pheromones [11], [24], [37], we were intrigued to discover that learning in young bees could be strongly affected by IPA. As IPA has been shown in previous studies to reduce sucrose sensitivity in bees [7], [9] differences in responsiveness to sucrose (the unconditioned stimulus) can influence learning performance [30], [32], [38], motivation for sucrose was carefully controlled in this study. Only bees that responded consistently to sucrose stimulations were used to examine learning performance. We felt justified in using this approach because IPA-treated bees that respond well to sucrose have been shown to have the same sensitivity to sucrose as controls [9]. Furthermore, there was no clear correlation between the number of bees responding to sucrose and the learning performance in each group. Moreover, in contrast to shifts in sucrose sensitivity [32], IPA altered learning, but not 1-hour memory. This suggests to us that IPA-induced changes in sucrose sensitivity do not account for IPA's ability to decrease learning in worker bees.

### IPA affects short-term but not medium-term appetitive memory

The discovery that IPA suppresses responses during the acquisition of short-term memory (STM), but not 1-hour memory retrieval is intriguing. This is especially so, as IPA's effects on appetitive learning have been shown to persist for at least 24 hours [9]. How can IPA's selective effects on learning performance be explained? We propose that physiological stress responses induced by IPA interfere with cellular and molecular processes that underlie STM retrieval, leading at the behavioral level to an apparent reduction in acquisition rate. Our results indicate that medium-term memory (MTM), on the other hand, is resistant to physiological changes induced by IPA.

In bees, as in many vertebrate and invertebrate species, short-, medium- and long-term memories differ not only in time course, but also in their molecular underpinnings [39]–[42]. In a series of elegant studies, Müller and colleagues showed that sucrose stimulation of the antenna causes a transient rise in the activity of the cAMP-dependent protein kinase A (PKA) in the antennal lobes of the bee brain [43]. They showed moreover, that a single pairing of an odor with sucrose extends the duration of PKA activation [44], a process that may potentially be slowed by IPA. The short-term memory that forms as a result of a single conditioning trial is unstable and highly susceptible to disruption [45]–[47]. However, multiple conditioning trials lead to the repeated updating of STM and the formation of stable, longer-lasting memories [42], [45], [48], [49]. Prolongation of PKA activity in the ALs has been shown to be critical for long-term memory formation in bees [44], whereas consolidation of MTM from STM requires ongoing neural activity and the formation of PKM, a constitutively activated form of protein kinase C [50]. Our results suggest the latter is resistant to modulation by IPA because irrespective of whether or not bees were exposed to IPA and/or QMP, bees of the same age exhibited strikingly similar response levels in the 1-hour memory test. It will be interesting in future studies to determine whether IPA-induced decrease of STM affects LTM performance and whether, as predicted, learning-related events in the antennal lobes of the brain are modulated by IPA. Mushroom bodies are intimately involved also in the formation and retrieval of associative olfactory memories [51], [52]. It is entirely possible that IPA-induced stress responses have an impact also on the functioning of learning-related circuits in these so called ‘higher’ centers of the brain.

### Stress reactivity in young bees is reduced by QMP

Our results show that young worker bees exposed to QMP respond less to the key component of alarm pheromone, IPA. The calming effect of the presence of the queen (and in particular her pheromonal bouquet) is well known to beekeepers: a hive that lost its queen will have more aggressive workers [53]. Her influence might help explain the low behavioral and olfactory responsiveness to alarm pheromone observed in young bees from queen-right colonies [11], as well as their lower levels of aggression [37]. Furthermore, QMP impairs aversive learning in young bees [15]. Taken together, these results suggest to us that QMP reduces stress reactivity in young worker bees.

QMP had no significant effect on appetitive learning performance in bees up to 6 days after their emergence as adults. However, around this time, bees appeared to go through a transition during which time they were more responsive to QMP and less responsive to IPA. Whether this is an artifact of rearing bees under laboratory conditions, or true also of bees in a normal queen right colony remains to be determined. What is clear from this study is that in young bees, inhibitory effects of IPA on learning performance are suppressed by QMP. Learning in cell cleaners, for example, which we predict to be between 1 and 5 days of age [10], [17]–[19], was not affected by IPA. Our results with bees of known age suggest that stress reactivity in cell cleaners is likely to have been inhibited by exposure to QMP within the colony.

### Interactions between IPA and QMP change with age

Responses of 8- and 16-day old bees to QMP were clearly different from those of bees less than 1 week old. In 8-day olds, QMP improved the learning performance of control bees, while IPA exposure failed to have any effect. This age group differed both from the young laboratory-raised bees and the naturally-reared pollen foragers and guards, and our results suggest that an important transition occurs at around 8-days of age (see also [15]). In the older age groups tested, the effects of IPA and QMP, as well as the interplay between these two pheromones, were markedly different to those observed in younger bees. Only older bees reared in presence of queen cues were affected by the alarm pheromone component, IPA. Importantly, pollen foragers and guards, whose learning performance was strongly affected by IPA exposure, came from a queen-right colony. We assume therefore that these bees would have been exposed to queen pheromone. Whether QMP exposure promotes reactivity to IPA at an older age remains to be determined. Age-related shifts in responses to QMP are well documented [10], [15], [54], [55], and correlate strongly with changes both in hormone titers [17], [31] and levels of gene expression in the brain [56]. Interestingly, QMP has been found to inhibit the synthesis of juvenile hormone [12], [13], a hormone that plays a significant role in honey bee behavioral development [17],[18]. While this may provide a clue as to how IPA-induced stress responses are blocked by QMP, the mechanisms involved remain unclear. One component of QMP (homovanillyl alcohol, HVA) has been found to suppress aversive learning in young worker bees [15], and to activate the D2-like dopamine receptor, AmDOP3 [57]. The functional consequences of AmDOP3 receptor activation by HVA have yet to be fully resolved, but it is possible that this QMP component is involved also in suppressing stress responses induced by IPA in young worker bees. The DOP3 receptor has recently been found to be expressed in the antennae [58], as well as in antennal lobes and mushroom bodies of the brain [59], [60]. As IPA and components of QMP are detected by olfactory receptor neurons housed in the antennae, QMP modulation of responses to IPA could potentially occur at this level [61], [62], but why this is age dependent remains unclear.

### Conclusions and predictions arising from this study

Our results show that IPA induces stress responses in young worker bees that can be blocked by QMP. Responses to these pheromones allow us to make several important predictions relating to the cellular and molecular mechanisms that support reward learning in honey bees. We predict firstly, that responses to acute stress modulate neural circuits involved in STM recall, but leave intact processes that underpin the formation and retrieval of MTM. We predict also that in young bees, QMP lowers responsiveness to IPA, a shift that should be detectable at the level of the antennal lobes. It will be interesting in future studies to test the strength of these predictions.
